# Determinants and the Moderating Effect of Perceived Policy Effectiveness on Residents’ Separation Intention for Rural Household Solid Waste

**DOI:** 10.3390/ijerph15040726

**Published:** 2018-04-11

**Authors:** Chuanhui Liao, Dingtao Zhao, Shuang Zhang, Lanfang Chen

**Affiliations:** 1 School of Economics and Management, Southwest University of Science and Technology, Mianyang 621010, China; zhangshuang@swust.edu.cn (S.Z.); Chenlanfang12333@163.com (L.C.); 2 School of Management, University of Science and Technology of China, Hefei 230026, China; box@ustc.edu.cn

**Keywords:** rural household solid waste, waste separation, extended theory of planned behavior, inducement policy, capacity building policy

## Abstract

Currently, villages “besieged with garbage” have become a serious problem in rural areas of China. Separation of rural residential solid waste (RRSW) is one of the main strategies for waste reduction. Although previous studies have analyzed the social and psychological motivations of residents’ separation intention for municipal solid waste (MSW), little attention has been paid to the situation in rural areas. This paper investigates key factors influencing rural residents’ separation intention, as well as analyzing the moderating effects of perceived policy effectiveness on the relationship between the determinants and the intention, using survey data of 538 rural residents in the province of Sichuan in China. The results show that all the proposed key factors influence the separation intention significantly. Furthermore, the policies were divided into two types and the moderating effects were tested for each type. The results show that the perceived effectiveness of both the inducement policy and the capacity building policy moderated the relationship between attitude and separation intention positively, while the perceived effectiveness of the inducement policy moderated the relationship between subjective norms and intention negatively. The findings provide insightful information for policymakers to design effective RRSW separation policies.

## 1. Introduction

Rural areas contain 42.65% of the population in mainland China as of the end of 2016 (National Bureau of Statistics of China. China Statistical Yearbook, 2017. Beijing: China Statistical Press, 2017 (in Chinese)). With the nation’s rapid economic development, rural areas in China are now confronted with serious environmental problems caused by a sharp increase of rural residential solid waste (RRSW) [1]. It is estimated that the annual growth rate of RRSW was 8–10% from 2000 to 2010 and reached 150 million tons as of 2015 [2]. The rising level of solid waste from rural households has led to “villages besieged with waste” becoming a serious problem in the rural areas in China, which has resulted in worsening environment that threatens the health of local residents [3].

RRSW separation is regarded as an efficient way to decrease the waste amount as well as reduce pollution from RRSW [1]. The governments of various countries have issued laws and implemented pilot campaigns for RRSW management and separation to minimize the waste. Moreover, incentives are provided by the governments to facilitate and encourage residents to participate in these campaigns, with either economic and/or social promotion measures [4]. In China, the government has proposed an RRSW management model named “household sorting, village collection, township transferring, and county treatment” in some pilot counties. Though this model has been implemented for over 18 years in some pilot counties, RRSW separation in rural areas is still in the infant stage around the country [1]. 

The implementation of RRSW management involves the participation of concerned stakeholders such as the residents, government, village committee, and local entrepreneurs (collecting, transportation, and incineration companies). The households or the residents are required to sort the waste and deliver the recyclables [5], and the governments are responsible for providing necessary facilities and incentives to encourage the residents to participate in the programs. In other words, the government and the residents should cooperate to ensure the implementation. This requires the policymakers to understand the residents’ psychological evaluations regarding the RRSW separation, as well as to investigate the obstacles that prevent the residents from taking appropriate action [6,7].

Previous researchers have deeply explored residents’ separation behavior regarding municipal solid waste (MSW) [7,8,9]. Most of the studies of separation behavior in MSW deal with psychological, social-demographic, and conditional characteristics. These factors have been confirmed to influence the residents’ separation behavior significantly [7,8,9]. Meanwhile, little research has been done on the separation of residential/household solid waste in rural areas in China. Current studies of RRSW management are mainly from the perspective of technology, public facilities planning and provision, and macro influencing factors [10,11]. In the studies investigating the determinants of RRSW management, macroeconomic characteristics and social capital are the main indicators involved [2,12]. As shown in studies of MSW separation, residents are the basis of the whole system, since the separation behavior should be carried out by the residents themselves. Hence there is a gap in investigating the rural residents’ psychological thoughts about RRSW separation, as well as their evaluation of the effectiveness of the policies provided. In the current article, perceived policy effectiveness (PPE) is defined as an individual’s favorable or unfavorable evaluation on the effects of these policies, i.e., whether the policies have clarity, adequacy, and facility to reach the target [13].

In this study, we choose rural areas as our target setting and do the investigation from the perspective of residents’ psychological factors and their perceived policy effectiveness (PPE), because of the following considerations. First, few published studies investigate the determinants of RRSW separation from the perspective of local residents. Most of the previous studies investigated determinants of RRSW reduction from the perspective of macroeconomic, geographic, and social capital factors [2,12]. There are few research efforts that investigate from the perspective of residents’ personal and psychological factors, compared to numerous fruitful outcomes of such efforts in the domain of MSW separation. Second, there are some differences in psychological and conditional factors between the urban and rural settings which do influence the residents’ separation intention and behavior accordingly [14]. Generally, rural residents are predicted to be more anthropocentric than their counterparts in conducting pro-environmental behavior [15,16]. In the context of China, Chen et al. [17] indicated that urban residents in larger cities were more likely to implement pro-environmental behaviors than those living in smaller cities. Thirdly, the demographic and conditional factors differ greatly between the urban and rural areas. The population density in rural China is much lower than in the urban areas. Hence the public infrastructure and communal facilities are better equipped in cities than in rural areas. Furthermore, the provision of RRSW management infrastructure and services is uneven across villages in China. It has been pointed out that the richer the area, the better the public infrastructure and facilities are equipped [18]. Considering the features of rural residents and differences in conditional factors, this paper intends to narrow the knowledge gap regarding determinants of personal psychological factors and PPEs in the domain of separation of RRSW.

Ajzen [19] developed the Theory of Planned Behavior (TPB) based on the Theory of Reasoned Action [20]. In the TPB model, behavior is determined by the behavioral intention, which is influenced by attitude, perceived behavioral control, and subjective norm [19]. TPB has been widely used in attitude-behavioral studies in different domains including the specific domain of MSW separation and recycling [7,8,9,10]. Although results of these studies vary in different contexts, the TPB model is generally accepted to be a suitable theory to predict pro-environmental intention and behaviors.

Despite the wide application of the TPB model, efforts to improve its predictive power have been conducted by researchers. According to Ajzen [19], adding conditional factors can improve the predictive power of the model and therefore some new constructs were added into the model such as moral norm, facilities, and behavioral consequence [4,6,21]. According to Bamberg and Schmidt [22], individuals would prefer to repeat behaviors that result in the most favorable consequences. Hence, past behavior is added to predict intention and future behavior [23,24]. Moreover, PPE is included in some studies. These studies assessed the individuals’ evaluation of the policy effectiveness and its direct effects on the behavioral changes [7,21,25]. Furthermore, perceived policy effectiveness is predicted and verified to moderate the relationship between TPB constructs and intention, thus influencing the strength and direction of the relationship between intention and actual behavior [21,24]. However, all these studies are based in the context of urban areas and little attention has been paid to rural context. Hence, in this study, we add two groups of constructs into TPB to test the direct effects of PPE and past behavior, as well as the moderating effects of PPE over separation intention of RRSW in the context of rural China.

This study aims to understand rural residents’ RRSW separation intentions in three steps. First, a framework was built to analyze the effects of psychological factors over RRSW separation intention using the original TPB model. Then the predictive power of the extended TPB model will be tested in step 2, by incorporating two groups of additional constructs (past behavior and perceived effectiveness of policy) into the TPB model. Lastly, efforts will be made to test whether PPE acts as a moderator between the constructs of the extended TPB model and intention. It is anticipated that through this research, the direct influence of residents’ personal concerns, past behavior, and PPE on RRSW separation intention, and the indirect influence of PPE, will be captured, which will provide well-founded suggestions to facilitate policy design and implementation.

The remainder of the paper is organized as follows. Section 2 deals with the literature review and research model development. Section 3 deals with the research method. In Section 4, data analysis and results are given. Conclusions, discussion, implication, and limitations are presented in Section 5.

## 2. Literature Review and Model Development

### 2.1. The Elements of the Original TPB

In TPB, there are three factors, i.e., attitude, subjective norm, and perceived behavioral control, which can influence behavioral intention. Attitude refers to the favorable or unfavorable perception an individual holds toward a particular behavior [19]. Attitude has been certified to be the strongest factor in predicting recycling and MSW separation intention and behavior [7,8,9,10]. Subjective norm refers to the perceived social pressure an individual feels from important others [19]. Studies have confirmed that subjective norm can influence behavioral intention positively [10]. In a society featuring collectivism, the influence from other people’s example and the expectation from important others to do a specific action may affect an individual’s behavioral intention and therefore change his/her behavior (Tonglet, 2004a). Perceived behavioral control (PBC) refers to the perceived ease or difficulty an individual feels toward a specific issue [19]. In the context of RRSW in China, perceived behavioral control includes perceptions of the availability of time, technology, and opportunity to conduct separation behavior. It is assumed that the more ability and opportunity an individual possesses, the higher the likelihood that the behavioral intention will come into force [26]. Based on this discussion, the following hypotheses are proposed.

**H1**: Attitude toward separation of RRSW will influence the separation intention positively.

**H2**: Subjective norm will influence the separation intention positively.

**H3**: Perceived behavioral control will influence the separation intention positively.

### 2.2. Past Experience and the Extended TPB Model

Past behavior is an action done previously and tends to predict intentions and future behavior [23,24,27]. It is predicted that past behavior should be regarded as a self-reinforcing process and act as an important reference for future behavior [28]. According to the self-cognitive theory, an individual can learn from his previous performance and make subsequent choices thereafter. Meanwhile, past behavior has been certified to influence individuals’ pro-environmental behavioral intention and behavior like recycling and MSW separation [28]. Therefore, if individuals with a history of RRSW separation may repeat this behavior and even form a habit in the subsequent session. Hence, the following hypothesis is proposed:

**H4**: Past experience will influence the separation intention of RRSW positively.

### 2.3. Perceived Policy Effectiveness and the Separation Intention

To make MSW separation universal, governments all over the world have issued laws, provided infrastructure, and given other incentives to promote residents’ waste separation intention and behavior. Wan and Shen [13] listed three types of policy tools: mandates, inducements, and capacity building. At present, the most utilized policy tools in RRSW separation in China are capacity building and inducements. Although the Chinese government has issued some regulations on RRSW management, most of them are concerning the standards of facilities and techniques in waste treatment, which have little influence on the residents’ side. Hence, the effectiveness of mandates is excluded from our consideration here. Concerning the capacity building in RRSW separation, both the governments and the village committees are responsible for the provision of infrastructure, propaganda, and education [2]. The inducement policies in this domain include waste charging, economic rewards, and public praise, varying in different rural areas. Based on the current situation of PPE in this domain, we classify the PPE into two types, the inducement and the capacity building policies, and test the direct and moderate effects of PPEs over separation intention.

Since government policies are regarded as motivations for RRSW separation, the residents are aware of these policies and would assess the effectiveness of these policies. Perceived policy effectiveness (PPE) is defined as an individual’s favorable or unfavorable evaluation on the effects of these policies, i.e., whether the policies are clear and adequate, and whether they facilitate reaching the target [13]. Previous studies analyzed the direct relationship between PPE and intention, and the results show that the policies have a significant influence on intention [13,24]. Moreover, the moderating effects of PPE are partly captured in some studies too [4,13].

Facilities for separation are indispensable in waste separation [13]. An individual will not take actual action without access to properly assigned facilities, even under a favorable attitude towards separation. Perceived lack of facilities has been found to influence the residents’ recycle intention directly in previous studies [4,6]. As for the inducement policies, waste charge, monetary bonus, and public praise are provided by the government and village committees. In the context of rural waste separation, people living in rural areas weigh anthropocentric consideration over eco-centric thoughts [15,16]. Hence the economic and social bonus policies contribute to higher participation, while the waste charging policies, acting as negative inducements, may have less significant effects. Here, items of the perceived effectiveness of waste charging are reverse coded so that we can combine the items of bonus and reversed charging into one construct. Hence, the following hypotheses are proposed:

**H5**: Perceived effectiveness of capacity building positively influences the RRSW separation intention.

**H6**: Perceived effectiveness of policy inducement positively influences the RRSW separation intention.

### 2.4. Moderating Effects of Perceived Policy Effectiveness

Apart from the direct effect on intention, PPE is predicted to have moderating effects over intention. Universally, policies are set up to facilitate separation and motivate residents to participate in RRSW separation. All such policies can be regarded as motivational devices, and motivations have proved to have moderating effects on the relationship between attitude and behavior [29]. Wan and Shen [13] predicted the moderating effects of PPE and further tested the effects using a survey of Hong Kong residents. In the case of MSW in urban China, Xu et al. [24] analyzed the moderating effects of PPE over the relationship between PPE and constructs of TPB in the context of the city of Hangzhou in China. However, all these analyses were conducted in the domain of MSW separation behavior and the PPE was measured by combining several types of policies into one constructor. Here in this study, we intend to measure the two types of PPE separately and analyze the moderating effects of each type respectively in the context of RRSW separation in China. 

#### 2.4.1. Moderating Effects of Perceived Effectiveness of Capacity Building 

Capacity building policies include the provision of facilities, public propaganda, and education, which are vital determinants in RRSW separation. It has been proven that capacity building is very important in promoting RRSW separation (Chen, 2010) and that a perceived low level of capacity building (e.g., perceived lack of facilities) may decrease the intention [4]. Thus, the PPE can be regarded as a moderator because PPE can influence the strength between the independent and dependent variables [30]. On one hand, improving the facilities’ condition and providing necessary propaganda and education can enhance the residents’ positive attitude to participate in separation [31]. On the other hand, if individuals find insufficient facilities and technology provision, they will not form the intention and take an actual action even with a high level of attitude [4]. At the same time, PBC is the perceived analysis an individual feels concerning the ease or difficulty of a specific behavior. Hence, the more capacity building measures are provided, the higher the perceived ease in conducting a behavior, thus leading to the positive moderating effects of PPE. Hence, the following hypotheses are proposed:

**H7a**: Perceived effectiveness of capacity building positively moderates the relationship between attitude and RRSW separation intention.

**H7b**: Perceived effectiveness of capacity building positively moderates the relationship between perceived behavioral control and RRSW separation intention.

A subjective norm is the example set and pressure imposed by important others, so that an individual can learn from them [32]. Theoretically, the effects of demonstration are stronger in the introductory stage [21]. When the subjective norm reaches a high level, which means almost all the people are taking part in the behavior, then the demonstration effect may diminish. That is to say, with the development of capacity building, especially with the deepening of public propaganda and education, more and more people may participate in the behavior, and then an individual will consider it as an ordinary issue and feel less perceived pressure from other people. The same situation occurs in the analysis of past behavior since past behavior is regarded as a self-reinforcing process and individuals can learn from their previous performance and then make subsequent choices. It has been pointed out that if a person forms a habit by repeating previous behavior, the link between previous behavior and future intention will become weak [28,33]. Based on this discussion, the following hypotheses are proposed:

**H7c**: Perceived effectiveness of capacity building policies negatively moderates the relationship between subjective norm and RRSW separation intention.

**H7d**: Perceived effectiveness of capacity building policies negatively moderates the relationship between past experience and RRSW separation intention.

#### 2.4.2. Moderating Effects of Perceived Effectiveness of Inducement Policies

Inducement policies refer to encouragements and incentives provided to stimulate a specific behavior [21]. In the context of RRSW separation in China, the most prevalent inducement policies used are waste charge, monetary incentives, and social praise. In most pilot villages of China’s RRSW separation program, in order to reimburse the cost of collection and transportation, the village committees require the villagers to pay a waste charge at the rate of ¥1.0 per capita per month (U.S.$1.8 per capita annually). Since this kind of waste charge serves as a disincentive (negative inducement), we reverse coded the related item in the data coding process. Monetary incentives are mainly daily necessities provided by the village committees to those who behave well in RRSW separation; this type of reward is preferred by most of the policymakers [34]. Social praise is public compliments for the best behaviors. Since rural people value anthropocentrism over eco-centrism when they analyze pro-environmental behavioral intention [15,16], we can predict that when more inducements are provided, there is more likelihood the rural residents will participate in the behavior, and the inducement policies can positively moderate the relationship between behavioral intention and attitude and perceived behavioral control. Hence, two hypotheses are proposed as follows:

**H8a**: Perceived effectiveness of inducement policy positively moderates the relationship between attitude and RRSW separation intention.

**H8b**: Perceived effectiveness of inducement policy positively moderates the relationship between perceived behavioral control and RRSW separation intention.

Though incentives policies (monetary or social incentives) can positively influence behavioral intention, it is predicted that these measures only work when the recycling rates are at a low level. When more and more residents participate in the separation activities, the effectiveness of incentives may decrease [35]. Li et al. [36] explored the incentives for food waste separation using a survey in Nanjing city, and found that material incentives (e.g., points exchanged for eggs) can motivate urban residents to participate in food waste separation when the separation rate is low. When more people take part in food diversion, separation norms come into being. Then the effect of monetary inducements decreases because people have formed the diversion habit [36]. In one study, when asked why they continue to sort food waste, 58% of the respondents answered that habit is the main consideration, and none mentioned monetary incentives. In another question about the most effective motivation in food waste sorting, habit was the dominant response [36]. Perceived effect of past experience may lead to repetition of the same behavior, resulting in habit formation, so we can draw the conclusion that inducements affect intention when there is a low level of norm, and its effectiveness decreases as the past behavior is repeated and finally the habit comes into being. Based on this reasoning, the following hypotheses are developed:

**H8c**: Perceived effectiveness of inducement policy negatively moderates the relationship between subjective norm and RRSW separation intention.

**H8d**: Perceived effectiveness of inducement policy negatively moderates the relationship between past experience and RRSW separation intention.

**Figure 1:**
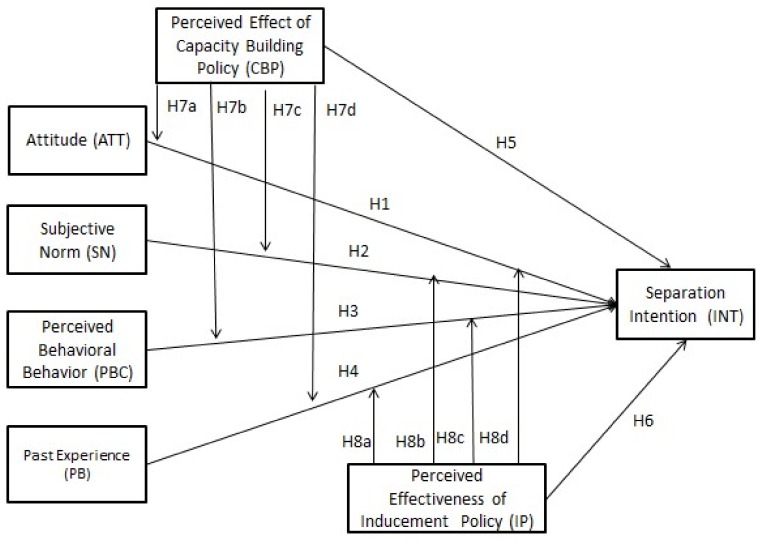
Research Framework and Hypotheses.

Based on the above-mentioned hypotheses, the research framework is depicted in Figure 1.

## 3. Method

### 3.1. Study Scope and Data Collection

Sichuan is one of the important agricultural provinces in China. In 2016, the total value of agriculture production of Sichuan amounted to ¥683 billion (about 106.7 billion U.S.D. at the exchange rate of 6.4), ranked No. 4 in 31 provinces (National Bureau of Statistics of China. China Statistical Yearbook, 2017. Beijing: China Statistical Press, 2017 (in Chinese)). There are about 42 million people living in the rural areas of the province as of the end of 2016, accounting for 50% of the population of Sichuan (Bureau of Statistics of Sichuan. Statistical Yearbook of Sichuan, 2017. Chengdu: China Statistical Press, 2017 (in Chinese)). Faced with a severe situation of “villages besieged with garbage”, the Sichuan government issued an “Implementation plan to strengthen the management of urban and rural residential waste in Sichuan province” in 2011. Although the outcome of the pilot campaign was satisfactory to some extent, the effect was confined to the pilot villages. A large number of villages were not involved in the pilot campaign, so the situation of RRSW separation is still a big problem for the local government. In 2017, seven counties and districts in Sichuan were chosen by the central government as the pilot counties of RRSW separation. In the current paper, we take Sichuan as the target area using a questionnaire survey to analyze the proposed hypotheses.

A random survey method was employed here. The sample was restricted to rural residents living in Sichuan. The survey was conducted from mid-July to mid-September of 2017. A face to face survey technique was used because of the difficulties doing online surveys in rural areas due to the comparatively low levels of education and internet service. The surveyors gave the questionnaire to the rural residents, and help was provided when the respondent had questions. If the respondent was an old person who could not read and write, the surveyor would read to the respondent word by word and fill in the questionnaire according to the respondents’ oral answers. Furthermore, gifts were provided for the participants to increase the response rate. The survey team distributed 700 copies of the questionnaire and got 594 copies back. Questionnaires with missing values and with identical answers on all different items were discarded. Finally, a sample of 538 useable responses was ready for further analysis, representing a response rate of 76%.

The demographic data of the sample are listed in Table 1. Among the respondents, 46.65% were male. In terms of age, 19.33% were below 20 and 48.7% were older than 60. The distribution of monthly income was relatively balanced: the monthly earnings of 5.76% were less than 1000 RMB, of 26.21% were 1001 to 1500 RMB, of 5.48% were from 1501 to 2000 RMB, and of 8.55% were above 2000 RMB. The education level of the respondents was relatively low, 53.35% had junior middle school education or below, and only 7.06% had a junior college education or had a bachelors’ degree or above. Generally speaking, the demographic data resembles the characteristics of rural residents in Sichuan.

### 3.2. Questionnaire Design and Measurements

The questionnaire was compiled by adopting items used in previous research on MSW separation and the TPB theoretical framework, with some items adapted according to the context of rural areas. The questionnaire was first drafted in English, then translated into Chinese. Two experts in environmental management and English teaching were invited to check the translation, and revision was made according to their feedback. Then we asked 20 university students from rural areas in Sichuan to send the questionnaire to their relatives. Feedback from the rural residents was collected and the questionnaire was adapted accordingly to make it easily understood by rural residents. In the end, a questionnaire containing 7 constructs with 23 items was ready for further use.

In the questionnaire, the constructs of the standard TPB model, i.e., attitude, subjective norm, and perceived behavioral control and intention were adapted from previous studies of Francis et al. [37] and Wan et al. [21]. Items of past behavior originated from the work of Wan et al. [38]. The new constructs, the perceived effect of capacity building and inducement policy, were taken from the study of Xu et al. [27] and Wan et al. [21]. As for the items of perceived effect of inducement policy, measurements were adopted from Wan et al. [27]. All these items were adapted according to the RRSW separation policies employed in Sichuan. A seven-point Likert scale was used to measure the items, with 1 representing “strongly disagree” and 7 denoting “strongly agree”. The Likert-scale method is a multiple-item scale and summed ratios are used to quantify constructs that cannot be directly measured. This method is popularly applied in research in the social sciences, marketing, and human environmental behaviors.

Questions concerning the demographic features of the respondents were also listed in the questionnaire. All the constructs and items are listed in Table 2.

## 4. Data Analysis and Results

Since all the data were based on self-reports from the same source at the same time period, common method bias (CMB) may exist here to threaten the validity of the research [39]. Hence, Harman’s one-factor test was used to test whether CMB existed or not. The results demonstrated that the items were divided into five constructs based on the criterion of the eigenvalues greater than 1. Together, these five constructs can explain 61% of the variance, with the first construct being 17.56%, conforming to the threshold value of 30% [40] and indicating that there was no serious CMB problem in this research.

Partial Least Squares (PLS), a common statistical approach to SEM, was used to conduct the analysis. According to Jöreskog and Wold [41], compared with covariance-based structural equation modeling, a distributional assumption of normality is not strictly required in PLS. Here the SPSS 23.0 (North Castle, NY, USA) and Smart-PLS 2.0 (Ringle, Christian M., Wende, Sven, and Will, Alexander (2005). SmartPLS 2.0.M3. Hamburg, Germany) were employed to implement the analysis.

### 4.1. Measurement Model

Two PLS models, the measurement model and the structural model, were tested.

Tests of the measurement model involve analyzing the convergent and discriminant validity of indicators. Cronbach’s alpha values and composite reliability values in construct reliability were employed to analyze the internal consistency of the indicators [42]. As shown in Table 3, the Cronbach’s α values ranged from 0.70 to 0.88, indicating suitable internal consistency between the items of each construct. Also, the composite reliability values ranged from 0.83 to 0.93, demonstrating that the items can represent each construct. Hence, we can draw a conclusion that the construct reliability is quite good.

Construct validity was employed to assess to what extent level the measurement scale can reflect the constructs, using convergent validity and discriminant validity [4]. Average variance extracted (AVE) was used and the results demonstrated that the AVE scores were among 0.63 to 0.87, better than the benchmark of 0.50 [42]. Discriminant validity was used to test the relationship between shared variance among the constructs by comparing the correlations to the discriminant validity. As demonstrated in Table 4, all the correlations between constructs were less than the square roots of AVE value, supporting the discriminant validity. Summarizing the above-mentioned discussion, we can draw the conclusion that adequate reliability and validity was achieved.

### 4.2. Structural Model Testing

The structural model was tested by analyzing the structural paths through examining the coefficients, t-statistics, and variance explained. Here, bootstrapping with 583 cases and 5000 subsamples was used to determine the path significance. Hierarchical regressions were conducted and the results are shown in Table 5. Model 1 includes constructs of original TPB, i.e., attitude, subjective norm and perceived behavioral control. In model 2, the additional constructs past behavior, perceived effectiveness of capacity building, and perceived effectiveness of inducement policy were added to test the explaining power of the extended TPB model. In the last model, the moderating effects of perceived effectiveness of two types of policies on the relationship between psychological constructs and intention were tested. Table 5 shows the results of the hierarchical regressions.

The results for model 1 indicate that all the constructs of original TPB model positively influenced the separation intention, conforming to previous studies [7,8,9]. Hence, H1, H2, and H3 were all supported. In model 2, all the added constructs were statistically significant with the separation intention. The path coefficient between separation intention and past behavior (b = 0.14, t = 3.31, *p* < 0.001), perceived effectiveness of capacity building policy and separation intention (b = 0.14, t = 4.23, *p* < 0.001), and separation intention and perceived effectiveness of inducement policy (b = 0.31, t = 6.71, *p* < 0.001) were all statistically significant and positive. This means that H4, H5, and H6 were all confirmed. Furthermore, the extended TPB model explained 75% of the variance in predicting the rural residents’ intention to separate RRSW, compared to 67% of the original TPB model (model 1). This indicates that the inclusion of past behavior, perceived effectiveness of capacity building policy, and inducement policy had increased the explanation of variance by 8% (∆R^2^ = 8%, *p* < 0.01), indicating that the extended TPB model had better predictive power. 

Moderating effects were tested in model 3. Of all the eight paths analyzing moderating effects, three paths yielded statistically significant results. The moderating effect of perceived policy effectiveness significantly increased the amount of variance explained for RRSW separation intention (∆R^2^ = 2%, *p* < 0.05). H7 predicted that perceived effectiveness of capacity building moderated the relationships between separation intention and all the independent variables, i.e., attitude, subjective norm, perceived behavioral control, and past behavior. The results in Table 5 indicate that only the moderating effect of H7a, i.e., the moderating effect of perceived policy effectiveness on the relationship between attitude and intention was statistically significant (b = 0.33, t = 2.28, *p* < 0.05). Hence, only H7a was supported, and H7b, H7c, and H7d were all rejected. The result can be explained that when a resident perceives a high degree of effectiveness of capacity building, the positive relationship between attitude and separation intention would be enhanced. 

H8 predicted that the perceived effectiveness of inducement policy moderated the relationship between the separation intention and all the four independent variables in the extended TPB model. Of the four moderating paths, two paths’ tests gave statistically significant results. The moderating effect of H8a, (i.e., the moderating effect of perceived effectiveness of inducement policy over the relationship between attitude and intention) was statistically significant (b = 0.87, t = 3.87, *p* < 0.001). Hence, H8a was supported. This implies that the greater the inducement policies, the stronger the positive relationship between attitude and separation intention (*p* < 0.001). Moreover, the moderating effect of H8c (i.e., the moderating effect of perceived effectiveness of inducement policy over the relationship between subjective norm and separation intention) was negatively significant (b = −051, t = 2.33, *p* < 0.05), indicating the certification of H8c. This indicates that if a resident perceives a high degree of effectiveness of inducement policies, the positive relationship between subjective norm and separation intention would diminish.

Of all the eight moderating paths, only three were significant. In the moderating effect of capacity building, only the coefficient of attitude → perceived effectiveness of capacity building → intention was significantly positive, which conforms to previous research results [29]. The result indicates that high perceived effectiveness of capacity building may facilitate the translation of positive attitude into RRSW separation. In the investigation of the moderating effect of perceived effectiveness of inducement policies, H8a was certified, which means there is a positive moderating effect on the relationship between attitude and intention. This conforms to the results of previous studies in that rural residents are more anthropocentric and less eco-centric than their urban counterparts in Trinidad and Canada [15,16]. These results, however, are contrary to the studies of MSW separation in the city of Nanjing, China, in which the urban residents think that the monetary incentive is not an important factor in their decision making [36]. Furthermore, the coefficient of the interaction term of subjective norm and the perceived effectiveness of inducement policy was negatively significant, conforming to the study of Wan et al. [21]. This means that the effect of subjective norm on separation intention would decrease with any increase in inducement policies. When a resident perceived a high degree of effectiveness of inducement policy, the positive relationship between subject norm and separation intention would diminish. In other words, strong subjective norms are associated with a stronger intention to separate waste, especially when the perceived effectiveness of the inducement policy was considered to be weak. This indicated that if the government provided effective inducement measures to encourage separation, the social influence and pressure would become less important, because when an individual is strongly motivated by inducement measures (monetary and/or public praises), the impact of the pressure from significant others will have a lower impact on the separation intention. Another reason explaining the negative moderating effect may be that the RRSW separation is still in its infant stage. It has been verified that subjective norm works best in the introduction stage and its effect diminishes with increases in participation. Governmental inducement policies induce more people to participate in the separation campaign, thus the relationship between subjective norm and intention diminishes.

## 5. Conclusions

This study investigates the effects of psychological factors, as well as the moderating effects of perceived policy effectiveness on RRSW separation intention using a survey of rural residents in Sichuan province in China. All the constructs, including the constructs of the original TPB model and the additional factors (past behavior, perceived effectiveness of capacity building, and perceived effectiveness of inducement policy) significantly influence the separation intention. Moreover, two types of perceived policy effectiveness (related to capacity building policy and inducement policy) are validated to partially moderate the relationship between all the independent variables in the extended TPB models and the intention. Specifically, on one hand, the perceived effectiveness of capacity building policy positively moderates the relationship between attitude and intention. On the other hand, the perceived effectiveness of inducement policies plays a positive and negative role between the attitude and intention, as well as between the subjective norm and intention.

This study contributes to the existing literature in two aspects. First, it is one of the first papers to explore the psychological determinants of RRSW separation intention in rural areas in China, compared with previous studies of RRSW that mainly dealt with macro social-demographic determinants [2,43]. Second, rather than measuring capacity building and inducement policies in a single constructor as was done in previous studies, the policies were divided into two types according to the current situation in rural areas of China [21,24]. The moderating effects of the perceived policy effectiveness of these two types over the extended TPB model were tested individually, providing targeted implications to the government and village committees.

The findings here have implications for the development of policies and programs. According to the results, residents will participate in RRSW separation when they have a positive attitude, subjective norm, perceived behavioral control, and past behavior. Hence, the Chinese government can provide more encouraging publicity about RRSW separation via TV programs, onsite demonstration, and posting to increase the number of residents who have a more positive attitude, subjective norm, and perceived behavioral control, as well as to change those who have less psychological motivation. Moreover, since the past behavior influences the intention positively, the village committee should publish the separation results of each family on the villages’ noticeboard, so the repeating of past behavior would gradually come to be a habit [28,33].

Since the residents’ perceived effectiveness of policy really impacts the intention to separate RRSW, the findings may provide considerations for the policymakers in designing and implementing separation policies. Firstly, according to the results for H8c, subjective norm becomes less important when the perceived effectiveness of inducement policies is high. Since the separation of RRSW is still not compulsory and remains at a low level in rural areas in Sichuan, the norm has not formed well. To strengthen the separation behavior, a high level of economic incentives is needed since rural residents are reported to be less eco-centric and more anthropocentric when they conduct pro-environmental behaviors [15,16]. Hence, the government and the village committees should provide more efficient economic incentives to the rural residents [1]. In our survey, 48.70% and 19.33% of the respondents are over sixty years old or less than 20 years old, because most of the young and middle-aged people go to work in cities, leaving the children and the older people in the rural areas [44]. Hence, the economic incentives should be designed to attract the young and the old: commodities such as life necessities and stationaries would be appropriate. Meanwhile, through the statistics on the results of the survey, most of the respondents were found to think highly of public praise. For the item IP3 “public praise can encourage me to separate RRSW”, 11% of all the respondents chose “1” or “2” (strongly disagree), 57% chose “6” and “7” (strongly agree), and 14% chose “4” (neutral). Hence, we can say that the rural residents think highly of public praise. This conforms to the social comparison theory that people strive to have stable and accurate appraisals of themselves [45]. This striving is more common in rural areas of China because the Confucian culture is better maintained in rural areas compared to urban areas. Face-saving is a key factor in rural residents’ behavior decisions and public praise can give more face and strengthen the pride and self-esteem of the residents [46]. Hence, the traditional methods of public praise should be used by the village committees, methods like the posting an honor roll on the open village affairs notice board. Secondly, H7a implies that the government and village committees should try to improve the provision of facilities and training courses for separation. For example, there should be sufficient sorting collection and a timely transportation network. In particular, wet waste like food waste should be transported in a short time to keep the facilities and settings hygienic. Moreover, the standardization of waste markets should be established. The informal recycling sector provided by scavengers and rag men was prevalent in rural areas in the past, but with the development of the economy and unstable prices of the recyclables, there are fewer rag men working in rural areas, leading to large quantities of excess unsalable recyclables. Hence, the government should provide subsidies to reimburse the collecting companies when the price of recyclables decreases, particularly for those recyclables of least market value, because an efficient collecting channel is vital for separation and the government facilitator can result in more effective recyclable collection [27].

This study contains several limitations that should be further explored in future research. First, the survey is restricted to the rural areas in the province of Sichuan with a sample of 538 residents. RRSW management varies due to the regional differences and unbalanced economic development around the country, so the results and application made in this paper are not universal; they are restricted to the specific areas of Sichuan province. Future studies should try to collect data from more provinces around China. Second, the current research focuses on intention instead of the behavior. Since a gap between the intention and behavior exists [20], in further study, more methods measuring the actual behavior should be used to investigate the policy effectiveness on actual behavior. Thirdly, the effects of socio-demographic features should be further studied. Social-demographic characteristics are predicted to influence the intention and behavior, as well as to moderate the relationship between constructs of the extended TPB model and intention in RRSW management [14,24]. Lastly, the effects of social incentives and economic incentives are diverse in different contexts so further study should pay attention to details of this aspect [46].

## Figures and Tables

**Table 1:** The social-demographic of the respondents.

Variable	Number of People	Percent (%)
**Age**		
<20	104	19.33
20–40	51	9.48
41–60	121	22.49
>60	262	48.70
**Gender**		
Male	251	46.65
Female	287	53.35
**Educational level**		
Junior middle school or below	287	53.35
Senior high school or below	213	39.59
Junior college or bachelors’ degree or above	38	7.06
**Income**		
<1000	31	5.76
1001–1500	141	26.21
1501–2000	320	59.48
>2000	46	8.55

**Table 2:** Constructs and measurement items.

Construct	Measurement Items	
Attitude (ATT)	ATT1	RRSW separation is good
	ATT2	RRSW separation is useful
	ATT3	RRSW separation is hygienic
	ATT4	RRSW separation is beneficial
Subjective norm (SN)	SN1	Most people who are important to me think I should separate RRSW
	SN2	My neighbors expect me to separate RRSW
	SN3	My family expect me to separate RRSW
Perceived Behavioral Control (PBC)	PBC1	I have plenty of opportunities to separate RRSW
	PBC2	I know how to separate my household waste
	PBC3	I have enough time to separate my household waste
Past behavior (PB)	PB1	I have separated my RRSW in the past 4 weeks.
	PB2	I have been separating my RRSW in the past 4 weeks.
	PB3	I have made waste separation behavior at home.
Perceived Effect of Capacity	CBP1	The separation facilities (bins, waste collection pools) provided by the government are sufficient to facilitate separation
Building Policy (CBP)	CBP2	The government provides clear guidelines and examples on separation
	CBP3	The government’s promotion clearly explains the benefits of separation
Perceived Effect of Inducement Policy (IP)	IP1	I agree to pay for the waste charge.
	IP2	The monetary incentives can encourage me to separate RRSW
	IP3	The public praise can encourage me to separate RRSW
	IP4	I agree to punish those who do not separate RRSW
Separation Intention (INT)	INT1	I intend to separate my household waste in the near future
	INT2	I will separate my household waste every time I have it for disposal
	INT3	I am willing to participate in the separation scheme in the near future

**Table 3:** Results of confirmatory factor analysis.

Construct	Items	Loadings	Cronbach’s Alpha	Composite Reliability	AVE
sAttitude (ATT)	ATT1	0.87	0.85	0.91	0.76
	ATT2	0.88			
	ATT3	0.87			
	ATT4	0.86			
liSubjective norm (SN)	SN1	0.88	0.82	0.89	0.74
	SN2	0.86			
	SN3	0.83			
Perceived behavioral control (PBC)	PBC1	0.73	0.70	0.83	0.63
	PBC2	0.81			
	PBC3	0.83			
Past behavior (PB)	PB1	0.84	0.83	0.90	0.75
	PB2	0.87			
	PB3	0.88			
Perceived effectiveness of inducement policy (IP)	IP1	0.94	0.84	0.93	0.87
	IP2	0.92			
	IP3	0.93			
	IP4	0.93			
Perceived effectiveness of capacity building policy (CBP)	CBP1	0.82	0.80	0.88	0.71
	CBP2	0.87			
	CBP3	0.83			
Intention (INT)	INT1	0.92	0.88	0.92	0.80
	INT2	0.93			
	INT3	0.84			

AVE average variance extracted.

**Table 4:** Means, standard deviation, and correlations.

Construct	Means	S.D.	ATT	IP	CBP	PB	INT	PBC	SN
ATT	4.52	1.21	**0.76**						
IP	4.47	1.25	0.59 **	**0.87**					
CBP	4.88	1.30	0.47	0.59 **	**0.71**				
PB	4.64	1.21	0.67 **	0.49	0.45	**0.75**			
INT	5.16	1.30	0.71 **	0.75 **	0.64 **	0.64 **	**0.80**		
PBC	5.40	1.25	0.46 **	0.53 **	0.60 **	0.47	0.59	**0.63**	
SN	4.78	1.19	0.65 **	0.69 **	0.60 **	0.58 **	0.74 **	0.56 **	**0.74**

The diagonal (bold) elements are the square roots of AVEs and the off-diagonal elements are the correlations among constructs. ** Shows significance at the 0.01 level. S.D. refers standard division.

**Table 5:** Testing of the main and moderating effects of RRSW separation intention.

Path	Model 1			Model 2			Model 3		
	Coefficients	T-Value	Hypothesis & Result	Coefficients	T-Value	Hypothesis & Result	Coefficients	T-Value	Hypothesis & Result
ATT → INT	0.37 ***	9.32	H1: Supported	0.22 ***	5.24		−0.57 **	2.71	
SN → INT	0.39 ***	10.18	H2: Supported	0.18 ***	4.12		0.45 **	2.85	
PBC → INT	0.21 *	6.03	H3: Supported	0.14 *	2.49		0.17 *	2.11	
PB → INT				0.14 ***	3.31	H4: Supported	0.16	1.18	
IP → INT				0.31 ***	6.71	H5: Supported	0.13	1.04	
CBP → INT				0.14 ***	4.23	H6: Supported	−0.40 ***	3.4	
ATT × CBP → INT							0.33 *	2.28	H7a: Supported
SN × CBP → INT							−0.02	0.37	H7b: NS
PBC × CBP → INT							0.02	0.03	H7c: NS
PB × CBP → INT							0.01	0.23	H7d: NS
ATT × IP → INT							0.87 ***	3.87	H8a: Supported
SN × IP → INT							−0.51 *	2.33	H8b: Supported
PBC × IP → INT							−0.21	1.43	H8c: NS
PE × PI → INT							−0.03	0.32	H8d: NS
R^2^	0.67			0.75			0.77		
f^2^	0.68								

* *p* < 0.05, ** *p* < 0.01, *** *p* < 0.001. NS refers to not supported.
